# Transgenic augmentation of erythroferrone in mice ameliorates anemia in adenine-induced chronic kidney disease

**DOI:** 10.1172/jci.insight.190018

**Published:** 2025-08-07

**Authors:** Brian Czaya, Joseph D. Olivera, Moya Zhang, Amber Lundin, Christian D. Castro, Grace Jung, Mark R. Hanudel, Elizabeta Nemeth, Tomas Ganz

**Affiliations:** 1 Center for Iron Disorders, Department of Medicine, and; 2 Department of Pediatrics, David Geffen School of Medicine at UCLA, Los Angeles, California, USA.

**Keywords:** Hematology, Nephrology, Bone marrow differentiation, Chronic kidney disease

## Abstract

Anemia is a common and disabling complication of chronic kidney disease (CKD). Current therapies can be burdensome, and full correction of anemia is limited by their cardiovascular side effects. New approaches that may offer additional therapeutic options are needed. We explored the antianemic effects of erythroferrone, an erythroid hormone that induces iron mobilization by suppressing the master iron-regulatory hormone hepcidin. In a preclinical murine model of adenine-induced CKD, transgenic augmentation of erythroferrone mobilized iron, increased hemoglobin concentrations by approximately 2 g/dL, and modestly improved renal function without affecting systemic or renal inflammation, fibrosis, or markers of mineral metabolism. This study supports the concept that therapeutic augmentation of erythroferrone is a promising approach for alleviating CKD-associated anemia.

## Abstract 2

In a preclinical murine model of chronic kidney disease, transgenic augmentation of erythroferrone mobilized iron, increased hemoglobin concentrations by approximately 2 g/dl, and modestly improved renal function.

## Graphical abstract

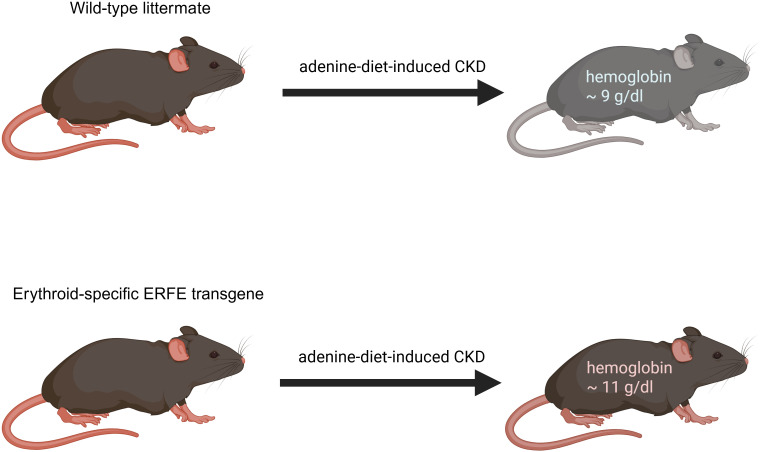

## Introduction

Chronic kidney disease (CKD) affects more than 10% of the population in the United States (1). Anemia, often severe if untreated, is a major morbidity in CKD and affects most of the patients with advanced CKD (2). The pathogenesis of this anemia is multifactorial, with mechanisms including impaired production of erythropoietin, dysregulated iron homeostasis, and the suppressive effects of inflammation and uremic toxins on erythropoiesis (3). Although the combination of erythropoietin derivatives and intravenous iron is effective in treating anemia (4), the logistical burdens for those patients who are not receiving regular hemodialysis have interfered with wider adoption of these therapies (5). Moreover, treatment with erythropoietin derivatives increases cardiovascular complications, and therefore is subject to limits that do not allow complete reversal of anemia. Orally administered prolyl hydroxylase inhibitors are also effective for treating anemia and more convenient for outpatient treatment, but they have not demonstrated improved cardiovascular risks compared with erythropoietin and i.v. iron (4). Additional treatment options are clearly needed (6).

Erythroferrone (ERFE) is a hormone produced by red cell precursors in the marrow and secreted into the blood stream (7). Its main documented effect is the transcriptional suppression of the hepatic iron-regulatory hormone hepcidin. Decreased hepcidin concentrations in blood then allow increased intestinal iron absorption and increased mobilization of iron from stores. In effect, physiologic augmentation of ERFE, such as occurs after blood loss or the administration of erythropoietin, facilitates the provision of iron for the increased requirements of intensified erythropoiesis.

To understand the effects of chronically elevated ERFE production, we generated transgenic mice with erythroid-specific *Erfe* overexpression driven by the β-globin promoter, which induces constitutive expression of *Erfe* in late-stage erythroid precursors — basophilic, polychromatic, and orthochromatic erythroblasts. This approach restricted transgene expression to bone marrow and yielded 3 independent transgenic lines with graded levels of *Erfe* overexpression (8). We observed a dose-dependent effect of ERFE on systemic iron loading, with the highest-expressing line (line-H) developing marked iron overload and a 1 g/dL increase in blood hemoglobin (Hb). Notably, line-H mice displayed significantly elevated *Erfe* transcript levels in bone marrow compared with WT littermates (mean –ΔCt: +2 vs. –8) and corresponding increases in serum ERFE concentrations (mean 200 ng/mL vs. below limit of detection) at both 6 and 16 weeks of age.

In the current study, we explored whether ERFE augmentation could be used as a treatment for CKD-associated anemia. We used the mouse model of adenine-induced nephropathy, in which excessive amounts of dietary adenine are administered and converted in vivo by xanthine oxidase to 2,8-dihydroxyadenine, which precipitates in the urine, causing tubulointerstitial kidney disease (9). A human genetic disorder, adenine phosphoribosyl transferase deficiency, causes a similar disease in humans (10). This mouse model recapitulates many characteristics of human CKD, including anemia, inflammation, and iron restriction (9, 11, 12).

## Results

**Figure 1:**
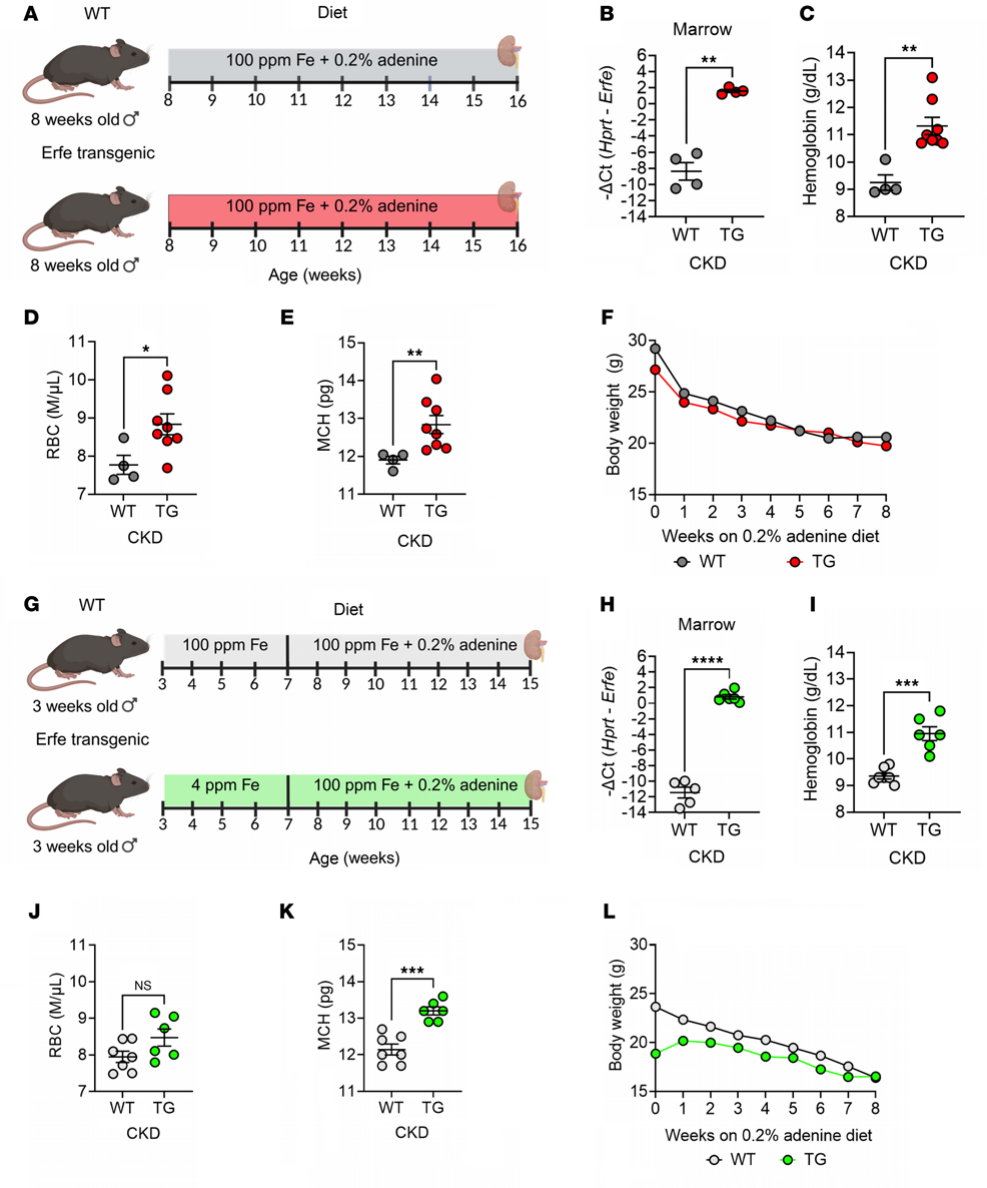
ERFE augmentation ameliorates CKD-associated anemia. (**A**) Pilot experimental design: 8-week-old WT littermate (*n* = 4) and ERFE-overexpressing transgenic (TG; *n* = 7) mice were fed 100 ppm iron (Fe) diet with 0.2% adenine for 8 weeks and then analyzed. (**G**) Final experimental design: Mice were weaned at 3 weeks, then WT (n = 7) mice placed on 100 ppm Fe diet and TG (*n* = 6) placed on 4 ppm Fe diet to prevent systemic iron loading prior to the initiation of adenine diet. At 7 weeks of age, WT and TG mice were placed on 0.2% adenine diet with 100 ppm Fe for 8 weeks and then analyzed. For both experiments, quantitative PCR (qPCR) analysis of (**B** and **H**) *Erfe* expression in bone marrow tissue (**C** and **I**), blood hemoglobin concentration, (**D** and **J**) RBC count, and (**E** and **K**) mean corpuscular hemoglobin (MCH) were determined. (**F** and **L**) Mice were weighed weekly. Data are mean ± SEM, analyzed by unpaired *t* test with Welch’s correction (2-tailed). *P < 0.05, **P < 0.01, ***P < 0.001, ****P < 0.0001. NS, not significant.

In an initial pilot experiment, we exposed line-H (high-expressing) *Erfe*-transgenic (TG) mice (8) and their wild-type (WT) littermates to a 0.2% adenine diet containing 100 ppm iron for 8 weeks (Figure 1A). As expected, both WT and TG mice developed anemia, appeared ill, and lost weight. Despite CKD-impaired erythropoiesis, TG mice exhibited markedly higher *Erfe* transcript levels in bone marrow compared with WT (mean –ΔCt: +2 vs. –8). Notably, TG mice maintained higher Hb levels (~2 g/dL), along with increased RBC count and mean corpuscular Hb (MCH), compared with WT controls (Figure 1, B–E). Moreover, despite TG mice starting with slightly lower body weights, weights remained relatively similar between genotypes throughout the course of disease, including at the time of harvest (Figure 1F). Although these findings are informative, we cannot differentiate the contribution of preexisting iron excess and higher Hb in TG mice at the start of adenine diet versus improved iron availability during the course of disease in this model, as TG mice have liver iron concentrations (LICs) 4.2-times higher than WT, and have 1 g/dL higher Hb concentrations by the age of 6 weeks (8).

To develop a more clinically relevant model, we noted that at weaning at 3 weeks of age TG mice had not yet developed iron overload, so that TG compared with WT mice had similar body weights, erythrocyte parameters, and slightly lower LICs (Supplemental Figure 1, A–F; supplemental material available online with this article; https://doi.org/10.1172/jci.insight.190018DS1). We observed that placing 3-week-old TG mice on an essentially iron-free (4 ppm) diet for 4 weeks (until the intended start of adenine diet) prevented iron accumulation, and these 7-week-old TG mice had even lower Hb concentrations, RBC count, MCH, LIC, and serum iron than their littermate WT mice (Supplemental Figure 1, G–L).

**Figure 2:**
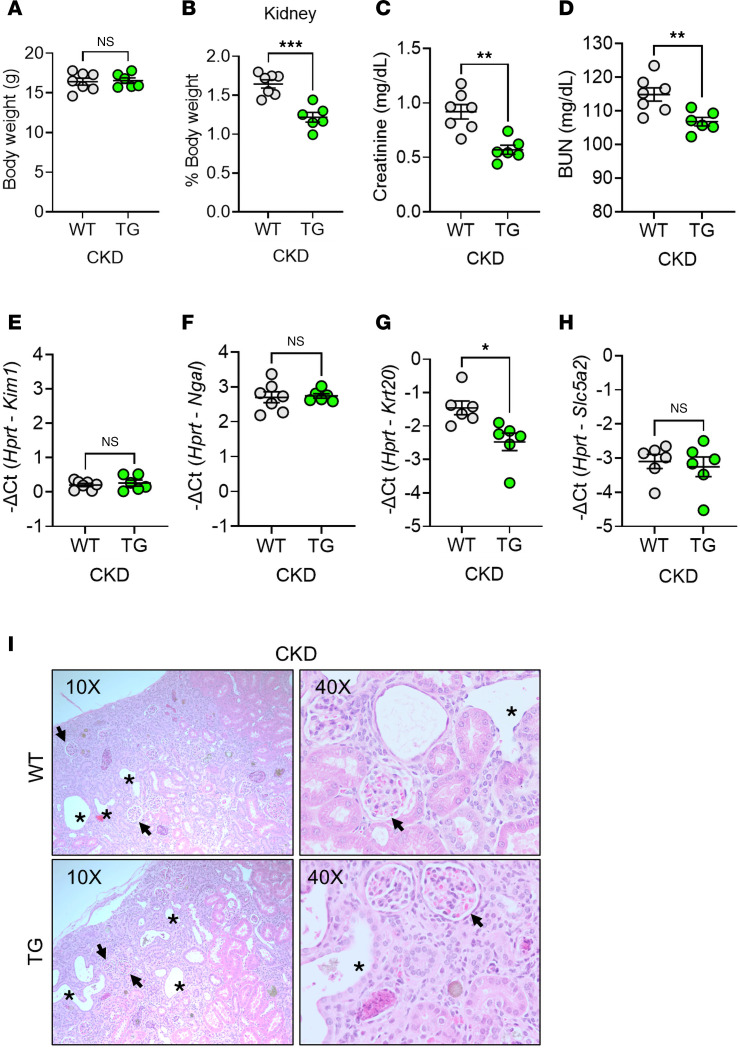
ERFE augmentation improves kidney function but does not alter injury markers in adenine-induced CKD. WT and TG mice from Figure 1G (*n* = 6–7 mice/group) were analyzed after 8 weeks on adenine diet. (**A**) Body weight, (**B**) kidney weight, (**C**) serum creatinine, (**D**) blood urea nitrogen (BUN). qPCR analysis of (**E**) *Kim1*, (**F**) *Ngal,* (**G**) *Krt20* and (**H**) *Slc5a2* expression in kidney tissue. (**I**) Representative H&E-stained kidney sections from WT and TG mice after 8 weeks on adenine diet (black asterisks, dilated tubule; black arrows, glomerular atrophy). Original magnification, ×10 (left) and ×40 (right). Data are mean ± SEM, analyzed by unpaired *t* test with Welch’s correction (2-tailed). *P < 0.05, **P < 0.01, ***P < 0.001. NS, not significant.

To induce CKD, parallel cohorts of 7-week-old iron-depleted TG mice and their 7-week-old WT littermates were placed on 0.2% adenine diet containing 100 ppm iron (Figure 1G). Despite more severe anemia and iron deficiency at the onset of adenine diet due to the iron depletion regimen, TG mice exhibited significantly higher Hb (~2 g/dL) and MCH compared with WT after 8 weeks on an adenine diet (Figure 1, H–K). This was accompanied by markedly elevated *Erfe* transcript levels in bone marrow of TG mice (mean –ΔCt: +1 vs. –11 in WT). Remarkably, the change in Hb between the pre- and postadenine groups was –4.7 g/dL for WT mice and +2.8 g/dL for TG mice (compare Supplemental Figure 1H and Figure 1I). Furthermore, despite starting at a lower body weight, TG mice ended up weighing the same as WT mice (Figure 1L, Figure 2A, and Supplemental Table 1).

As we previously reported (8), developmental exposure to elevated ERFE leads to mildly elevated blood urea nitrogen (BUN) levels in TG mice, falling within the upper physiological range for mice, potentially reflecting reduced nephron number and/or impaired nephron development. However, normal kidney morphology and absence of baseline proteinuria indicate preserved glomerular filtration barrier integrity despite reduced kidney function. After 8 weeks of adenine diet, TG mice still exhibited smaller kidneys than WT mice (Figure 2B). However, while renal function was impaired in both WT and TG mice (higher BUN compared with normal [~20–30 mg/dL]; ref. 8), both BUN and creatinine were lower in TG than WT mice, indicating significant amelioration of CKD by transgenic ERFE (Figure 2, C and D). The expression levels of certain renal injury markers (*Kim1*, *Ngal*, and *Slc5a2* [SGLT2]) were similar in WT and TG mice, whereas *Krt20* was lower in TG, consistent with some degree of protection from kidney injury (Figure 2, E–H). The histological appearance of kidney tissue sections was comparable between WT and TG mice, with similar frequencies of tubular dilation and glomerular atrophy, consistent with adenine-induced tubulointerstitial injury (Figure 2I).

**Figure 3:**
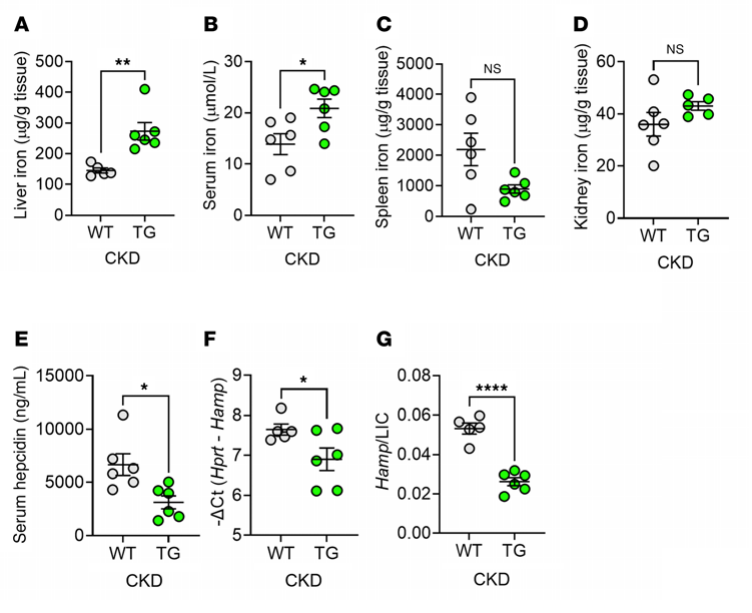
ERFE augmentation lowers hepcidin levels to enhance iron mobilization in adenine-induced CKD. WT and TG mice from Figure 1G (*n* = 6–7 mice/group) were analyzed. (**A**) Liver iron concentration (LIC). (**B**) Serum iron. (**C** and **D**) Tissue iron concentrations in (**C**) spleen and (**D**) kidney. (**E**) Serum hepcidin levels. (**F**) Liver hepcidin mRNA expression. (**G**) Liver hepcidin mRNA/LIC. Data are mean ± SEM, analyzed by unpaired *t* test with Welch’s correction (2-tailed). *P < 0.05, **P < 0.01, ****P < 0.0001. NS, not significant.

To investigate the mechanism of anemia amelioration by transgenic ERFE, we examined systemic iron parameters (Figure 3). After 8 weeks of adenine diet, TG mice had higher LICs (reflective of iron stores), higher serum iron, and nonsignificantly lower splenic iron than WT mice (Figure 3, A–C). This indicates that TG mice had increased iron absorption (reflected by LIC) and possibly increased iron mobilization from red pulp macrophages (reflected by spleen iron). Kidney iron did not significantly differ between TG and WT mice (Figure 3D). Serum hepcidin and hepatic hepcidin expression were lower in TG than in WT mice, despite higher LIC, and the ratio of hepatic hepcidin expression to LIC was markedly lower in TG versus WT mice (Figure 3, E–G), consistent with the documented hepcidin-suppressive effect of ERFE (7, 8). Lower hepcidin concentrations and improved iron availability for erythropoiesis in TG compared with WT mice would be expected to increase Hb concentrations and RBC hemoglobinization, as was observed (Figure 1).

The effects of ERFE on kidney injury could be complex. On one hand, amelioration of anemia would be expected to improve oxygen delivery and benefit the energy-intensive functional and repair processes in the kidney. On the other hand, ERFE’s mechanism of action depends on inhibition of BMP signaling involved not only in the regulation of hepcidin transcription (13–15) but also in kidney development and repair (16–18).

**Figure 4:**
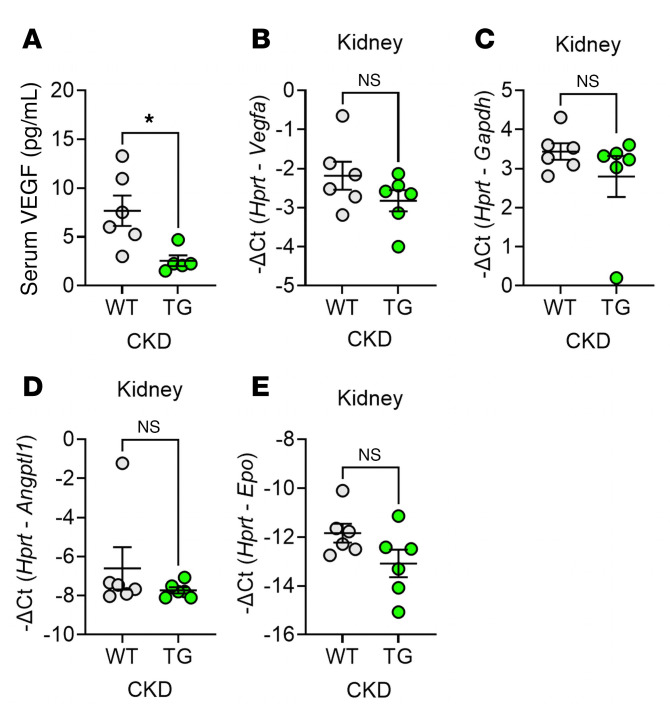
ERFE augmentation enhances systemic oxygenation without significantly impacting kidney oxygenation in adenine-induced CKD. WT and TG mice from Figure 1G (*n* = 6–7 mice/group) were analyzed. (**A**) Serum VEGF levels. (**B**–**E**) Kidney mRNA concentrations by qPCR for (**B**) V*egfa*, (**C**) *Gapdh*, (**D**) *Angptl1*, and (**E**) *Epo*. Data are mean ± SEM, analyzed by unpaired *t* test with Welch’s correction (2-tailed). *P < 0.05. NS, not significant.

We first examined the effects of transgenic ERFE on hypoxia-sensitive processes in adenine-induced CKD mice (Figure 4). Serum VEGF was lower in TG than in WT mice, indicative of improved tissue oxygenation in TG mice (Figure 4A). In the kidney at this advanced disease stage, the expression of *Vegfa*, *Gapdh*, *Angptl1*, and *Epo* all trended lower in TG than in WT mice but the differences were small and not statistically significant (Figure 4, B–E).

We next analyzed the expression of kidney and liver BMP-regulated genes (Supplemental Figures 2 and 3). In the kidney, expression of *Id1*, *Id2*, *Id4*, *Bmp2*, and *Bmp7* was similar between TG and WT mice, while *Smad7* was significantly lower in TG mice (Supplemental Figure 2, A–F). In the liver, despite increased hepatic iron in TG mice on an adenine diet (Figure 3A), expression of BMP target genes (*Id1*, *Id2*, *Atoh8*, and *Smad7*) remained unchanged (Supplemental Figure 3, A–D), indicating that liver BMP/SMAD signaling was inappropriately low relative to iron loading. This likely reflects disrupted iron sensing driven by CKD-associated inflammation (19). Supporting this notion, normalization of gene expression to LICs revealed reduced *Smad7* expression in TG mice compared with WT (Supplemental Figure 3E), consistent with ERFE-mediated suppression of liver BMP signaling.

**Figure 5:**
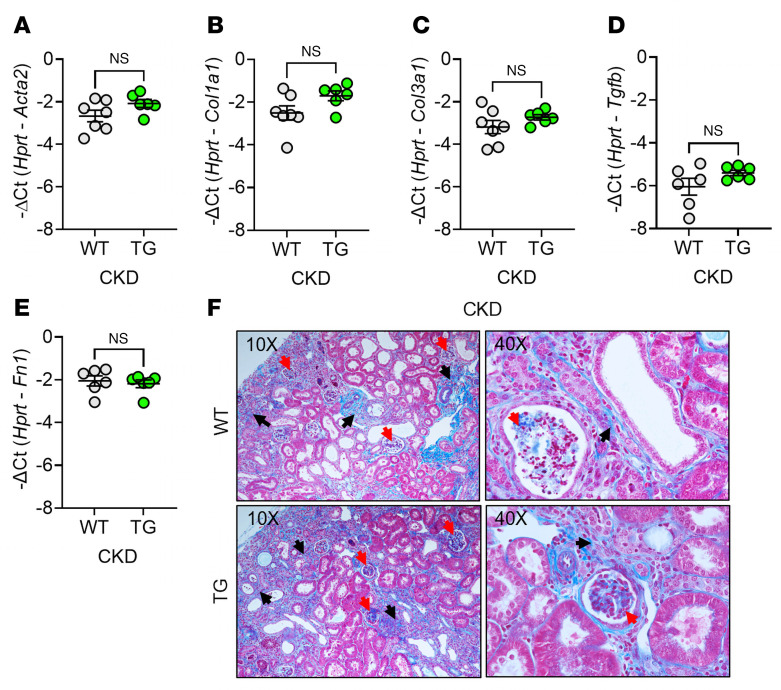
ERFE augmentation does not affect kidney fibrosis in adenine-induced CKD. In WT and TG mice from Figure 1G (*n* = 6–7 mice/group), kidney tissues were analyzed by qPCR for (**A**) *Acta2*, (**B**) *Col1a1*, (**C**) *Col3a1*, (**D**) *Tgfb1,* and (**E**) *Fn1*. (**F**) Representative Masson’s trichrome–stained kidney sections from WT and TG mice after 8 weeks on adenine diet (black arrows, interstitial fibrosis; red arrows, glomerulosclerosis). Original magnification, ×10 (left) and ×40 (right). Data are mean ± SEM, analyzed by unpaired *t* test with Welch’s correction (2-tailed). NS, not significant.

The expression of renal fibrosis markers could potentially be affected by BMP signaling, but levels of *Acta2* (α-smooth muscle actin), *Col1a1* and *Col3a1* (type I and III collagen α1 chains), *Tgfb* (TGF-β1), and *Fn1* (fibronectin) were not significantly changed (Figure 5, A–E). Masson’s trichrome staining revealed comparable degrees of interstitial fibrosis and glomerulosclerosis in WT and TG mice, with kidney sections displaying similar overall histopathological features (Figure 5F). In the aggregate, although there was a detectable suppression of a sensitive BMP signaling–related marker in the kidney (Supplemental Figure 2D), there was no appreciable effect on renal fibrosis.

**Figure 6:**
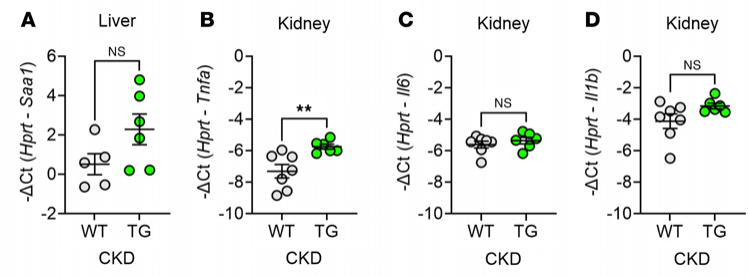
ERFE augmentation does not substantially change mRNA markers of liver or kidney inflammation in adenine-induced CKD. WT and TG mice from Figure 1G (*n* = 6–7 mice/group) were analyzed by qPCR for (**A**) *Saa1* expression in liver tissue and (**B**–**D**) *Tnfa, Il6*, and *Il1b* expression in kidney tissue. Data are mean ± SEM, analyzed by unpaired *t* test with Welch’s correction (2-tailed). **P < 0.01. NS, not significant.

Inflammation is an important driver of both anemia (20) and kidney injury (21). We therefore examined whether transgenic ERFE affected systemic and organ inflammation elicited by 2,8-dihydroxyadenine crystals in the kidney and by the subsequent impairment of renal function. We found no significant differences between WT and TG mice in plasma concentrations of key inflammatory cytokines TNF-α, IL-1β, IL-6, IL-1α, IFN-γ, or IL-10 (Supplemental Figure 4, A–F). The expression of the acute-phase reactant and hepatic inflammatory marker *Saa1* and that of renal cytokines *Il6* and *Il1b* was similar between WT and TG mice with adenine-induced CKD, while renal expression of *Tnfa* was mildly increased in TG compared with WT mice (Figure 6, A–D). Overall, ERFE overexpression did not have a substantial effect on systemic or renal inflammation.

Dysregulated mineral metabolism in CKD is closely linked to iron dyshomeostasis and contributes to adverse patient outcomes, including bone diseases and cardiovascular disease, in part through greatly increased FGF23 levels (22, 23). Linked to FGF23 excess, left ventricular hypertrophy is a common and serious complication of CKD (24, 25). Accordingly, we analyzed (Supplemental Figure 5) the effect of ERFE overexpression on serum phosphate and iFGF23 (biologically active form) concentrations, as well as markers of mineral metabolism, including *Slc34a1* and *Slc34a3* (encoding sodium-dependent phosphate transporters 2A and 2C, also known as NaPi-2a and -2c), *Cyp27b1* and *Cyp24a1* (encoding the enzymes that respectively generate and break down the active form of vitamin D, 1,25-dihydroxyvitamin D3), and *Kl* (encoding Klotho, the renal coreceptor for iFGF23). No significant effects of transgenic ERFE overexpression were observed (Supplemental Figure 5, A–G), except for a small increase in the expression of the phosphate transporter *Slc34a1* that mediates phosphate reabsorption in the renal tubular brush border. This increased expression may reflect the improved kidney function in TG mice (Figure 2).

## Discussion

In this proof-of-concept study in mice, we present evidence that *Erfe* overexpression mitigates anemia in a preclinical mouse model of adenine-induced CKD. At the end of the study, Hb was 2 g/dL higher in TG compared with WT mice, and the difference was even greater when Hb change is considered relative to the baseline preadenine group (–4.7 g/dL for WT mice and +2.8 g/dL for TG mice). These Hb improvements represent clinically significant increases. Notably, no overt adverse effects were observed in TG mice relative to WT during CKD progression.

As shown in this study and a preceding report (8), the primary pathway impacted by transgenic overexpression of ERFE is iron homeostasis. The effect of ERFE augmentation is to increase LIC and reduce iron content in the spleen. These effects are attributable to ERFE’s well-documented ability to suppress hepcidin, by inhibiting hepatic BMP signaling (13–15), the key pathway regulating hepcidin transcription in hepatocytes (26). Hepcidin suppression results in enhanced dietary iron absorption and mobilization of iron from macrophage stores. Clinically, these findings suggest that in patients with CKD, ERFE could promote the availability of sequestered dietary or medicinal iron for erythropoiesis, and enhance erythropoietic response to therapeutic erythropoietin.

Apart from animal studies, genetic evidence in humans indicates that ERFE would also mobilize iron and stimulate erythropoiesis in humans, as human *ERFE* gene intronic variant rs13007705-T leads to increased transferrin saturation, Hb, and MCH in genome-wide association studies (https://www.ebi.ac.uk/gwas/variants/rs13007705).

Improving CKD-associated anemia is linked to multiple clinical benefits, including enhanced kidney function and reduced cardiovascular risk (27, 28). In our adenine nephropathy model, ERFE overexpression modestly improved kidney function, as reflected by lower BUN and serum creatinine levels. The improvement in kidney function is noteworthy because it occurs despite the developmental effect of ERFE that results in smaller kidneys and slightly higher BUN in this mouse model. TG mice also exhibited reduced proximal tubular injury (Figure 2 and Supplemental Figure 5) and less severe systemic hypoxia (Figure 4) compared with WT. Although the precise mechanisms by which ERFE modulates kidney dysfunction remains unclear, our findings suggest that the improved kidney function in TG mice may be an indirect consequence of improvement of anemia, increased oxygen delivery, and reduced renal hypoxia. Supporting this hypothesis, constitutive ERFE-knockout mice with CKD (EKO-CKD) treated with the HIF prolyl hydroxylase inhibitor vadadustat — an agent used to treat CKD-associated anemia — displayed significantly lower BUN and serum creatinine levels compared with vehicle-treated EKO-CKD mice (29). Nonetheless, further studies are needed to determine whether ERFE exerts direct renoprotective effects, particularly at the level of tubular cell function.

This study has several limitations. First, we used only male mice, as female mice are relatively resistant to adenine-induced crystal formation and CKD development (30). However, ERFE is not expected to act differently in females; our prior work in non-CKD mice demonstrated that ERFE overexpression exerts similar effects across sexes (8). Second, although the adenine-rich diet is a widely accepted model for studying CKD and its complications, elevated systemic levels of adenine can exert pleiotropic effects beyond the kidney. Notably, adenosine — a metabolite of adenine — has been shown to impair erythropoiesis by reducing erythroid precursor proliferation and survival (31). However, such effects would likely impact both genotypes similarly in our study. Moreover, the pattern of improvement in complete blood count in ERFE-augmented mice indicates that the increase in Hb concentration is predominantly driven by increased MCH, consistent with increased availability of iron, rather than increased RBC number. Third, despite our chosen strategy to model a clinically relevant transition from iron deficiency to iron repletion when currently available treatments are initiated for CKD-related anemia, fully disentangling the effects of ERFE from iron status is inherently complex. Lastly, transgenic augmentation of ERFE in utero impacts embryonic development in TG mice, leading to smaller baseline kidney weight. This developmental effect may lead to underestimation of potential renal benefits of ERFE treatment for CKD-associated anemia. For proof of concept, we chose the transgenic approach, as administration of currently available recombinant ERFE may induce an inflammatory response due to protein aggregation characteristic of the C1q/TNF-related protein family (7). Further technical development will be needed to overcome this limitation or find alternative means of augmenting ERFE. Nonetheless, our findings provide valuable insights into the therapeutic potential of ERFE.

Other approaches specifically targeting the hepcidin/ferroportin pathway in CKD are in various stages of clinical development, and include monoclonal antibodies targeting the positive regulators of hepcidin, including BMP6 (32) and hemojuvelin (33), and monoclonal antibodies against the hepcidin receptor and iron transporter ferroportin (32). In the current study, we employed a common preclinical mouse model of CKD to verify the concept that the endogenous erythroid hormone ERFE can be used therapeutically to ameliorate anemia.

## Methods

### Sex as a biological variable.

The study employs male mice, as CKD in rodents presents a more robust and consistent phenotype in males than in females (34, 35). Similar results are expected for female mice.

### Mice.

Mice were maintained in a ventilated rodent-housing system in temperature-controlled environments (22°C–25°C) with a 12-hour light/dark cycle, and allowed ad libitum access to food and water. Unless specified, mice received a standard diet (PicoLab Rodent Diet 20, 5053; irradiated, 185 ppm iron). TG mice were generated as previously detailed (8), and the line with highest *Erfe* expression (line-H) was used in the current study. These mice were maintained on a C57BL/6J background.

To evaluate the therapeutic impact of ERFE on CKD-related complications, WT littermates and TG mice were administered a specialized adenine-rich diet with adequate iron content (0.2% adenine, 100 ppm iron; TD.210096, Envigo) for 8 weeks to induce CKD (Figure 1A). To account for the potential confounding variable of incipient chronic iron overload already apparent at approximately 6 weeks of age in TG mice, they were initially placed on an iron-deficient diet (4 ppm iron; TD.80396, Envigo) at weaning for 4 weeks (Supplemental Figure 1G). One cohort of TG mice was analyzed at this point to establish iron and hematological parameters at the onset of the study, and another cohort was switched to the 0.2% adenine diet with adequate iron (100 ppm; TD.210096, Envigo) for an additional 8 weeks (Figure 1G). Age-matched WT mice followed a similar protocol, receiving an iron-adequate diet (100 ppm iron; TD.200065, Envigo) for 4 weeks after weaning, with one cohort euthanized at this point and another transitioning to the 0.2% adenine-rich, iron-adequate diet for 8 weeks (Figure 1G). Upon completion of these experimental timelines, mice were euthanized under 2.5% isoflurane anesthesia.

### Hematologic parameter and iron-related measurements.

Complete blood counts were analyzed using a HemaVet blood analyzer (Drew Scientific). Serum iron and tissue non-heme iron levels were measured through colorimetric quantification following the manufacturer’s instructions (157-30, Sekisui Diagnostics). To reduce variability from regional iron distribution, whole liver, spleen, and kidney tissues were ground in liquid nitrogen before sampling.

### Serum chemistry.

Serum hepcidin concentrations were determined by ELISA as previously described (7). BUN (DIUR-100, BioAssay Systems), and serum creatinine (80350, Cayman Chemical) concentrations were measured by colorimetric quantification. Intact bioactive FGF23 (iFGF23) levels were assessed by ELISA (60-6800, QuidelOrtho). Serum phosphate levels were assessed by colorimetric quantification (830125, Thermo Fisher Scientific). Assays were performed following the manufacturers’ protocols.

### Cytokine and chemokine measurements.

Serum cytokine and chemokine levels were analyzed by the UCLA Integrated Molecular Technologies Core using a multiplex bead-based immunoassay (Millipore Milliplex Cytokine/Chemokine 32-plex Kit on Luminex FlexMap3D), following established protocols (36).

### Histopathology.

Kidney tissues were fixed in 10% neutral buffered formalin for 24 hours at room temperature, rinsed twice in distilled water, and stored in 70% ethanol until processing. Tissues were paraffin-embedded, sectioned at 4 μm thickness, and subjected to staining with hematoxylin and eosin (H&E) for morphological analysis or Masson’s trichrome for fibrosis evaluation, performed by the UCLA Translational Pathology Core Laboratory. Slides were viewed and imaged using a Nikon Eclipse E600 microscope with SPOT Basic (SPOT Imaging) image software.

### Quantitative PCR analysis.

Frozen mouse tissues were homogenized in TRIzol Reagent (15596018, Thermo Fisher Scientific) and total RNA was extracted using chloroform. Employing a 2-step reaction method, 500 ng of total RNA was reverse transcribed into cDNA using the iScript cDNA Synthesis Kit (1708896, Bio-Rad). Quantitative real-time PCR was conducted with 20 ng of cDNA, SsoAdvanced Universal SYBR Green Supermix (172-5272, Bio-Rad), and sequence-specific primers (Supplemental Table 2). Samples were assayed in duplicate with a CFX Connect Real-Time PCR detection system (Bio-Rad). Relative gene expression was normalized to *Hprt* levels. Data are presented as –ΔCt.

### Statistics.

Data organization, scientific graphing, and determination of the statistical significance of differences between experimental groups were performed by using GraphPad Prism (version 10.2.3). Data are presented as individual values representing the mean ± SEM. Statistical differences between groups was determined by unpaired *t* test with Welch’s correction (2-tailed). Statistical test, number of animals per group, and *P* value are indicated in each figure panel and legend. A *P* value of less than 0.05 was considered significant.

### Study approval.

All animal studies were approved by the UCLA Institutional Animal Care and Use Committee, and were performed in accordance with the NIH *Guide for the Care and Use of Laboratory Animals* (National Academies Press, 2011).

## Author contributions

BC designed and performed experiments, analyzed data, and wrote the manuscript. JDO, MZ, AL, CDC, and GJ assisted with experiments. MRH and EN assisted with data analysis and interpretation. TG supervised the project, assisted with data interpretation, and wrote the manuscript. All authors discussed results, read and contributed edits to the manuscript and approved the final version.

## Supplementary Material

Supplemental data

Supporting data values

## Version 1 08/07/2025

In-Press Preview

## Version 2 09/23/2025

Electronic publication
