# Supplementary material for: Transgenic augmentation of erythroferrone in mice ameliorates anemia in adenine-induced chronic kidney disease
Source: JCI Insight. 2025 Aug 7;10(18):e190018. doi: 10.1172/jci.insight.190018 (PMC12487854; doi:10.1172/jci.insight.190018)
Supplement: Supplemental data [file jciinsight-10-190018-s085.pdf]

Supplemental Figure and Data

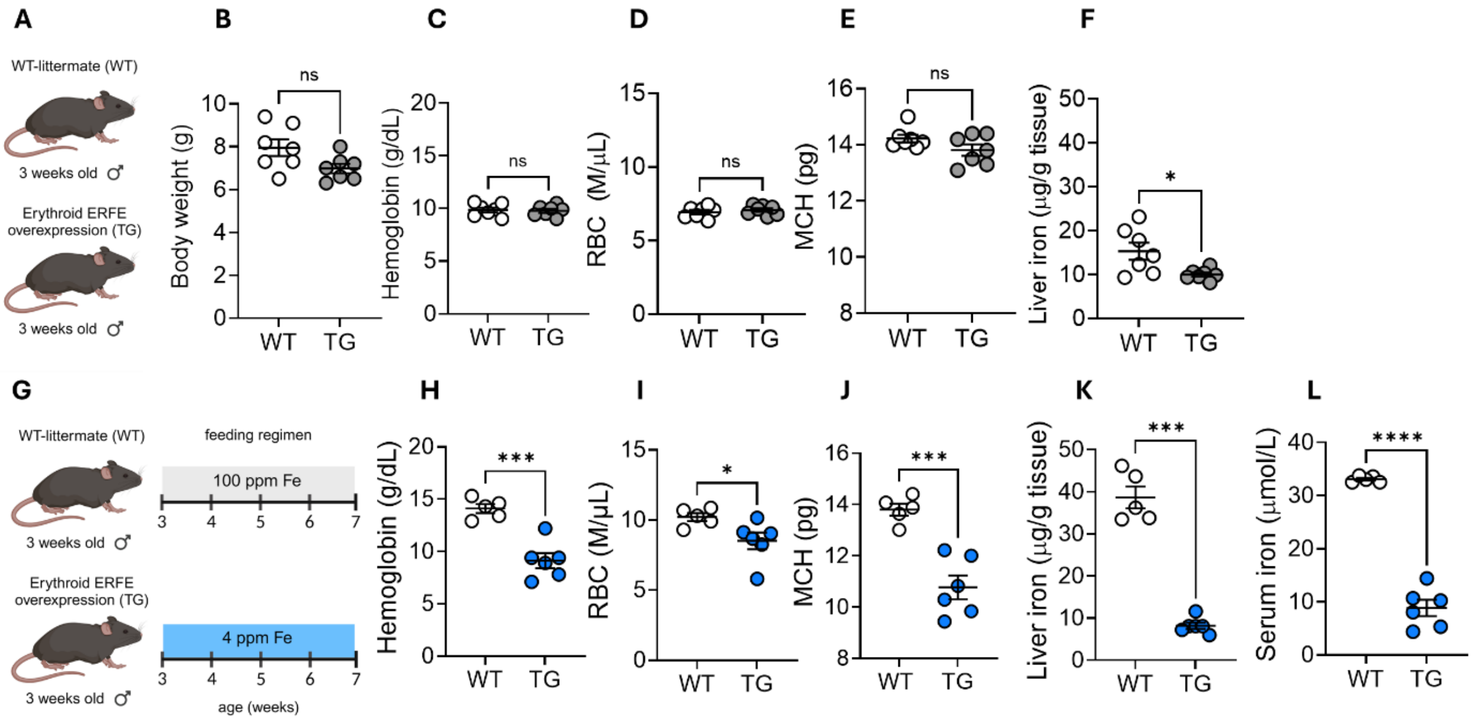

Figure S1. Experimental design development. (**A**) WT and TG (n= 7 mice/group) mice at weaning (3 weeks of age), (**B**) body weight, (**C**) hemoglobin concentrations, (**D**) RBC count, (**E**) MCH, (**F**) liver iron concentration (LIC). (**G**) At weaning (3 weeks of age), WT and TG (n=5-6/group) mice were placed on 100 ppm Fe diet and 4 ppm Fe diet respectively, and analyzed after 4 weeks for (**H**) hemoglobin concentration, (**I**) RBC count, (**J**) MCH, (**K**) liver iron concentration (LIC) and (**L**) serum iron concentration. Data are mean ± SEM, analyzed by unpaired-*t*-test with Welch's correction (two-tailed). ***P < .001; *P < .05; ns = non-significant.

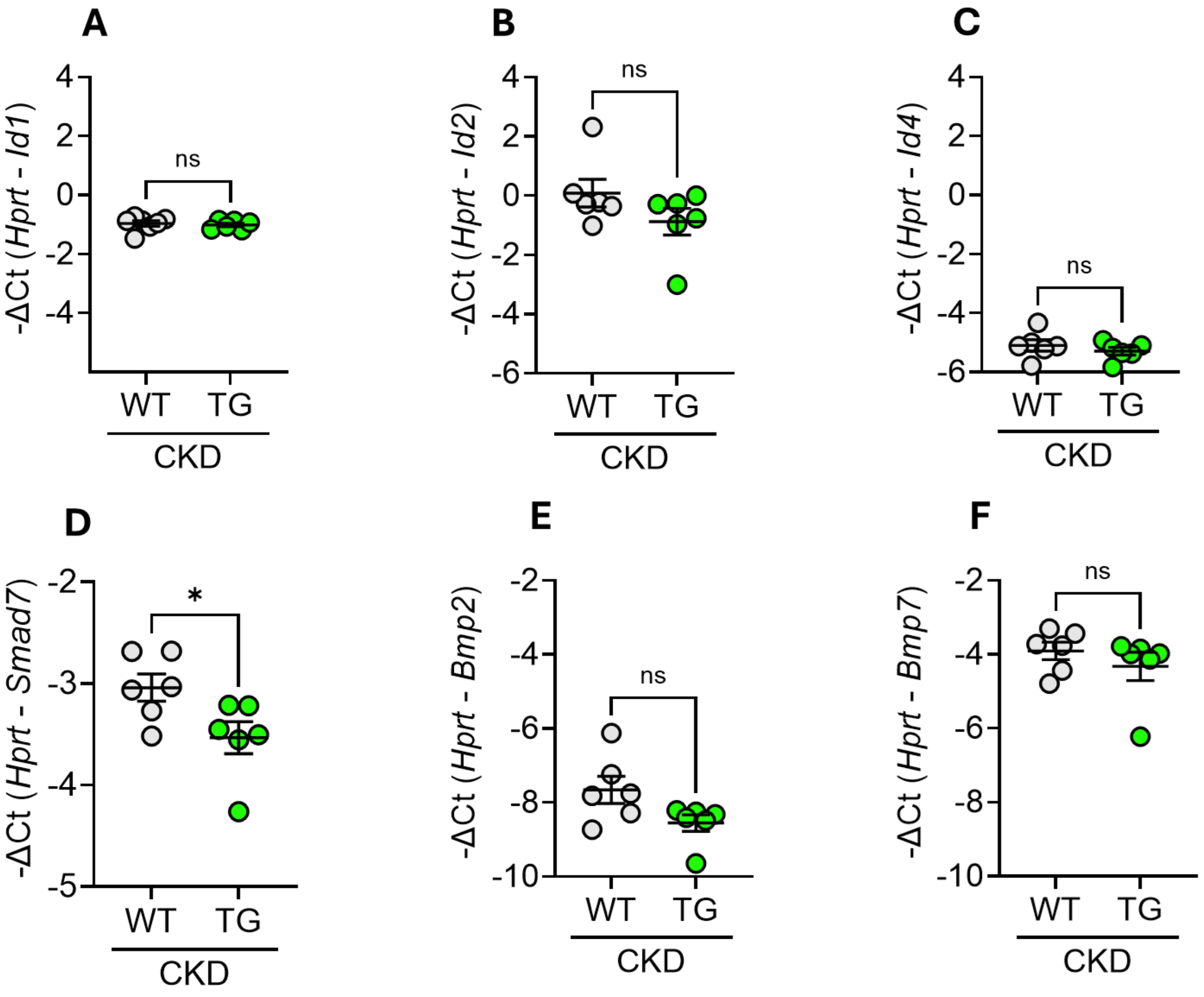

Figure S2. ERFE augmentation results in unchanged or mildly suppressed kidney BMP signaling in adenine-induced CKD. WT and TG mice from Figure 1G (n=6-7 mice/group) were analyzed by qPCR of kidney tissue for (**A**) *Id*1, (**B**) *Id2*, (**C**) *Id4*, (**D**) *Smad7*, (**E**) *Bmp2* and (**F**) *Bmp7*. Data are mean ± SEM, analyzed by unpaired-*t*-test with Welch's correction (two-tailed). *P < .05; ns = non-significant.

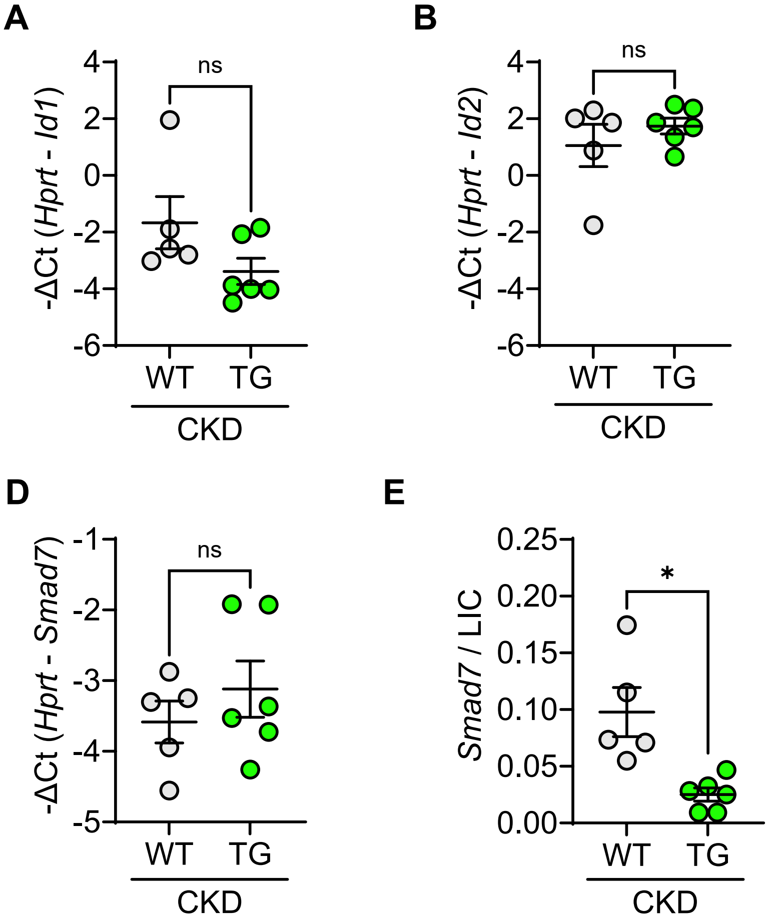

Figure S3. ERFE augmentation results in unchanged or mildly suppressed liver BMP signaling in adenine-induced CKD. WT and TG mice from Figure 1G (n=5-6 mice/group) were analyzed by qPCR of liver tissue for (**A**) *Id*1, (**B**) *Id2*, (**C**) *Atoh8*, (**D**) *Smad7*, (**E**) liver *Smad7* mRNA/LIC. Data are mean ± SEM, analyzed by unpaired-*t*-test with Welch's correction (two-tailed). *P < .05; ns = non-significant.

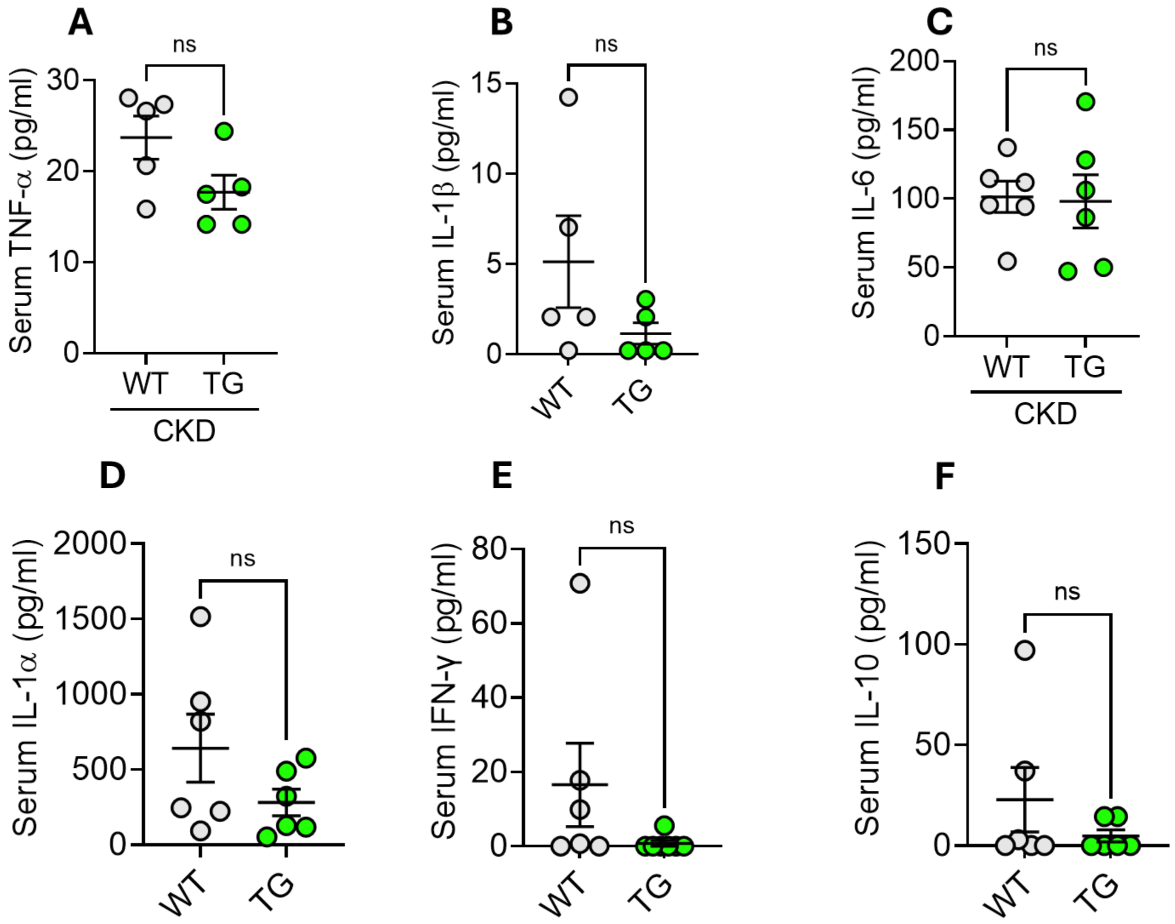

Figure S4. ERFE augmentation does not impact systemic inflammation in adenine-induced CKD. WT and TG mice from Figure 1G (n=6-7 mice/group) were analyzed by serum 32-plex cytokine/chemokine assay for (**A**) TNFα, (**B**) IL-1β, (**C**) IL-6, (**D**) IL-1α, (**E**) IFNγ, and (**F**) IL-10. Data are mean ± SEM, analyzed by unpaired-*t*-test with Welch's correction (two-tailed). ns = non-significant.

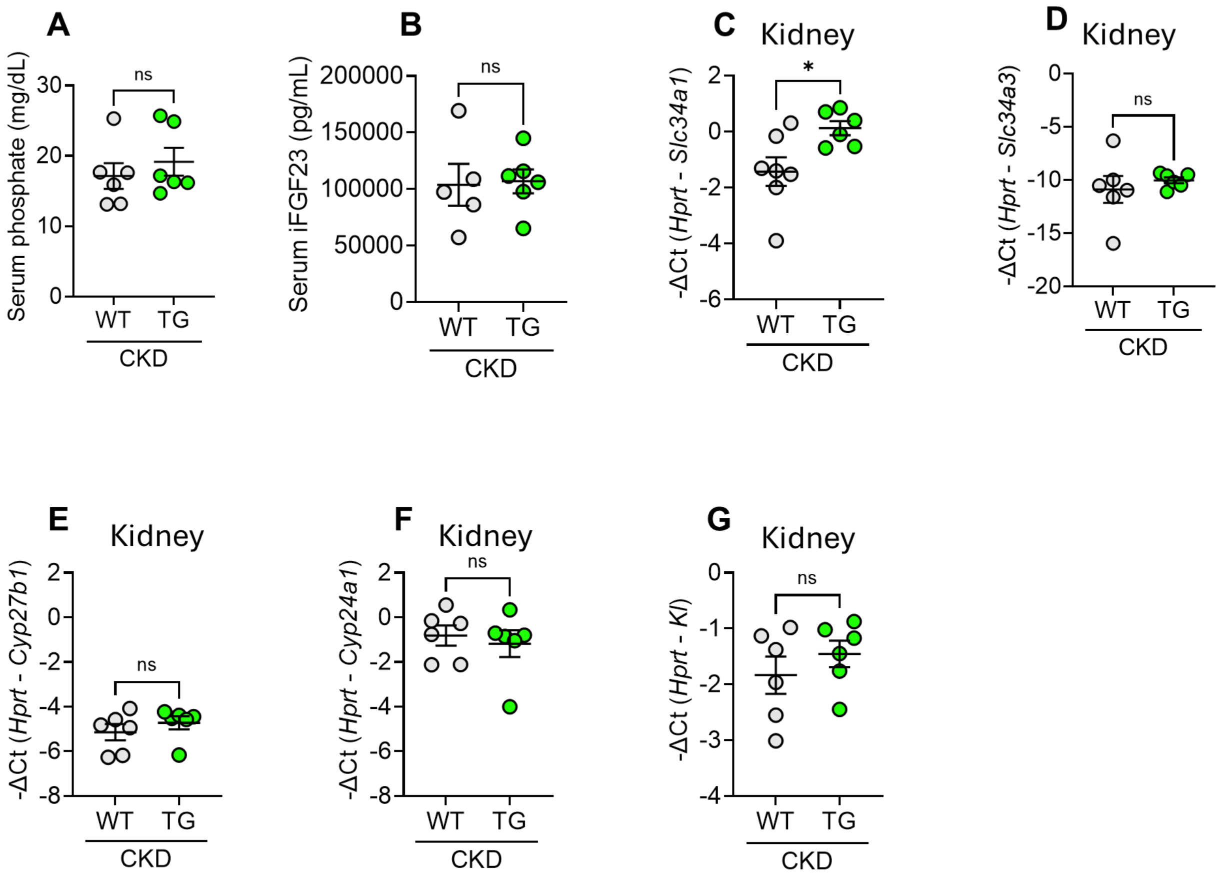

Figure S5. ERFE augmentation does not alter markers of mineral metabolism in adenine-induced CKD. WT and TG mice from Figure 1G (n=6-7 mice/group) were analyzed. Measured parameters include (**A**) serum phosphate, (**B**) serum intact iFGF23, and qPCR analysis of kidney tissue for (**C-G**) *Slc34a1* and *Slc34a3 (*encoding sodium-dependent phosphate transporters 2A and 2C), *Cyp27b1* and *Cyp24a1 (*encoding the enzymes that respectively generate and break down the active form of vitamin D, 1,25-dihydroxyvitamin D3) and *Kl* (encoding Klotho, the renal coreceptor for iFGF23). Data are mean ± SEM, analyzed by unpaired-*t*-test with Welch's correction (two-tailed). *P < .05; ns = non-significant.

Supplemental Table 1: Body and organ weights of wildtype (WT) and ERFE-TG (TG) mice receiving a 0.2% adenine diet as described in Figure 1G.

	WT + 0.2% adenine diet	TG + 0.2% adenine diet
Starting body weight (g)	23.7 ± 0.7	18.9* ± 0.8
Final body weight (g)	16.4 ± 0.4	16.5 ± 0.4
Wet liver weight (mg)	691.4 ± 32.4	711.5 ± 31.3
Wet spleen weight (mg)	44.1 ± 3.5	54.8* ± 2.4
Wet kidney weight (mg)	262.5 ± 12.1	207.3* ± 13.3
Liver to body weight (%)	3.78 ± 0.2	4.32* ± 0.1
Spleen to body weight (%)	0.2 ± 0.02	0.32* ± 0.02
Kidney to body weight (%)	1.6 ± 0.1	1.2* ± 0.1

Values are expressed as mean ± SEM. Comparison between groups was performed in form of an unpaired-t-test with Welch's correction, N=6/group; *$p \leq 0.05$.

Supplemental Table 2. Oligonucleotides used as sequence specific primers in qPCR analyses.

Gene	Species	Orientation	Primer Sequence (5' – 3')
Acta2	Mus musculus	Forward<br>Reverse	GTC CCA GAC ATC AGG GAG TAA<br>TCG GAT ACT ACT TCA GCG TCA GGA
Col1a1	Mus musculus	Forward<br>Reverse	ATG GAT TCC CGT TCG AGT ACG<br>TCA GCT GGA TAG CGA CAT CG
Col3a1	Mus musculus	Forward<br>Reverse	GAC CAA AAG GTG ATG CTG GAC AG<br>CAA GAC CTC GTG CTC CAG TTA G
Krt20	Mus musculus	Forward<br>Reverse	CAA CGG ATC GGA CCT GTT TG<br>AGC GCA CTT TTT CTA GGT AGT TT
Fn1	Mus musculus	Forward<br>Reverse	GAT GTC CGA ACA GCT ATT TAC CA<br>CCT TGC GAC TTC AGC CAC T
Hamp	Mus musculus	Forward<br>Reverse	GAG CAG CAC CAC CTA TCT CC<br>TTG GTA TCG CAA TGT CTG CC
Hprt	Mus musculus	Forward<br>Reverse	CTG GTT AAG CAG TAC AGC CCC AA<br>CGA GAG GTC CTT TTC ACC AGC
Il1b	Mus musculus	Forward<br>Reverse	TGC CAC CTT TTG ACA GTG ATG<br>TGA TGT GCT GCT GCG AGA TT
Il6	Mus musculus	Forward<br>Reverse	CTC TGG GAA ATC GTG GAA AT<br>CCA GTT TGG TAG CAT CCA TC
Havcr1/Kim1	Mus musculus	Forward<br>Reverse	CGA GTG GAG ATT CCT GGA TGG<br>GGA CGT GTG GGA ATC TCT GG
Slc5a2/Sglt2	Mus musculus	Forward<br>Reverse	TGG GCT GGA TAT TTG TCC CGA<br>CAA ACC GCT TCC GCA GAT ACT
Vegfa	Mus musculus	Forward<br>Reverse	ACA TTG GCT CAC TTC CAG AAA CAC<br>GGT TGG AAC CGG CAT CTT TAT C
Lcn2/Ngal	Mus musculus	Forward<br>Reverse	GTC CCC ACC GAC CAA TGC<br>ATT GGG TCT CTG CGC ATC C
Gapdh	Mus musculus	Forward<br>Reverse	CCA ATG TGT CCG TCG TGG ATC T<br>GTT GAA GTC GCA GGA GAC AAC C
Angptl1	Mus musculus	Forward<br>Reverse	GAC AAT TCA CTC GAA CTT TCC CA<br>CCT TGT TGC CAT CTT CAG CAT T
Saa1	Mus musculus	Forward<br>Reverse	ACA CCA GCA GGA TGA AGC TAC T<br>GAG CAT GGA AGT ATT TGT CTG AGT
Epo	Mus musculus	Forward<br>Reverse	TCT ACG TAG CCT CAC TTC ACT<br>ACC CGG AAG AGC TTG CAG AAA
Tgfb	Mus musculus	Forward<br>Reverse	ATA CGT CAG ACA TTC GGG AAG CAG TG<br>AAT AGT TGG TAT CCA GGG CTC TCC G
Klotho/Kl	Mus musculus	Forward<br>Reverse	TGA TGT CGT CCA ACA CGT AGG CTT<br>GCA AAG TGC TCA ACT GGC TAA GGT
Tnfa	Mus musculus	Forward<br>Reverse	CCC TCA CAC TCA GAT CAT CTT CT<br>GCT ACG ACG TGG GCT ACAG

Id1	Mus musculus	Forward<br>Reverse	CCT AGC TGT TCG CTG AAG GC<br>CTC CGA CAG ACC AAG TAC CAC
Id2	Mus musculus	Forward<br>Reverse	ATG AAA GCC TTC AGT CCG GTG<br>AGC AGA CTC ATC GGG TCG T
Id4	Mus musculus	Forward<br>Reverse	CCG CCC AAC AAG AAA GTC AG<br>CCA GGA TGT AGT CGA TAA CGT G
Smad7	Mus musculus	Forward<br>Reverse	GGG CTT TCA GAT TCC CAA CTT<br>AGG GCT CTT GGA CAC AGT AGA
Bmp2	Mus musculus	Forward<br>Reverse	TCA AGC CAA ACA CAA ACA GC<br>AGC CAC AAT CCA GTC ATT CC
Bmp7	Mus musculus	Forward<br>Reverse	CAT CGT CCA GAC ACT GGT TCA C<br>TCG AAG TAG AGG ACA GAG ATG GC
Slc34a1/NaPi2a	Mus musculus	Forward<br>Reverse	GGC TCC AAC ATT GGC ACT ACC A<br>ACC ACA GTA GGA TGC CCG AGA T
Slc34a3/NaPi2c	Mus musculus	Forward<br>Reverse	GCG GTA TTA CCA GCA ACA CCA C<br>TGT CCT CCT CTG GAG ATG CTG A
Cyp27b1	Mus musculus	Forward<br>Reverse	TTC GGC TTT GGC AAA CGG AGC T<br>GGC TTG ATA GGA AGA GCA CCT G
Cyp24a1	Mus musculus	Forward<br>Reverse	GCT CCT TCA AAA GGA CAC AGA GG<br>CGC TTG CCA CAC TTT GGT GTT G
Fam132b/Erfe	Mus musculus	Forward<br>Reverse	ATG GGG CTG GAG AAC AGC<br>TGG CAT TGT CCA AGA AGA CA
